# Knee Pain and Future Self-Reliance in Older Adults: Evidence From a Community-Based 3-Year Cohort Study in Japan

**DOI:** 10.2188/jea.JE20100118

**Published:** 2011-05-05

**Authors:** Yuji Nishiwaki, Takehiro Michikawa, Mutsuko Yamada, Norihito Eto, Toru Takebayashi

**Affiliations:** Department of Preventive Medicine and Public Health, School of Medicine, Keio University, Tokyo, Japan

**Keywords:** joint diseases, activities of daily living, mortality, cohort studies, aged

## Abstract

**Background:**

Although knee pain is common in older persons and can cause ambulatory limitation, its impact on self-reliance has rarely been examined in Japan, particularly in a community setting. The aim of this 3-year cohort study was to investigate the association of knee pain with dependence in activities of daily living (ADL) and mortality in community-dwelling older Japanese adults.

**Methods:**

In 2005, presence of knee pain was assessed by a home visit survey of 1391 older adults aged 65 years or older (participation proportion = 97.3%). A total of 1265 participants who were ADL-independent at baseline were followed for 3 years, and information on outcomes, namely death and dependence in ADL, was collected.

**Results:**

Participants who always had knee pain were more likely to become dependent in ADL than those who reported no knee pain (multivariate-adjusted OR, 1.98; 95% CI, 1.03–3.83); however, always having knee pain was not associated with mortality or a composite outcome of ADL dependence and death. Further analyses of each component of ADL dependence revealed that knee pain was associated with a need for assistance at home (long-term care eligibility, bathing, dressing, and transferring), but not with institutionalization.

**Conclusions:**

The participants were highly representative of the target population and the rate of follow-up was almost perfect (99.4%). The results suggest that knee pain is associated with future dependence in ADL, particularly a need for assistance at home.

## INTRODUCTION

Knee pain is common in older adults.^1^^,^^2^ However, there is considerable discordance between knee pain and radiographic knee osteoarthritis (OA).^3^^,^^4^ The proportion of individuals with both knee pain and radiographic OA was reported to range from 15% to 76%, and, among those with radiographic knee OA, the proportion of individuals with pain ranged from 15% to 81%.^3^ It is usually the perception of pain, rather than the presence of pathology, that troubles patients, and pain is the principal reason for seeking treatment.^1^ In addition, assessment of perceived pain is more straightforward, less expensive, and faster than measuring radiographic changes of OA, which is obviously advantageous for an epidemiologic study that hopes to attain a high participation proportion of the target population.

The self-reliance of older adults is an increasingly important public health concern as the global population ages. Knee pain can cause ambulatory limitation.^2^ Therefore, it is an important factor in loss of independence in activities of daily living (ADL), and even death, because ambulation is a good predictor of mortality in older persons.^5^ Many studies of the association between knee OA, including knee pain, and dependence in ADL have been conducted.^6^^–^^8^ In Japan, however, evidence is limited. Watanabe et al reported that mobility was significantly lower in patients with knee OA than in control subjects,^9^ and Kondo et al investigated risk factors associated with knee pain and functional limitation.^10^^,^^11^ In both these studies, the subjects were hospital-based patients with knee OA. However, a recent population survey in the United Kingdom found that among 3023 adults aged 50 years or older who reported knee pain within the previous year, only 33% reported visiting their general practitioner for this complaint during the same period.^12^ Therefore, community-based studies are desirable to produce less biased estimates of the association between knee pain and dependence in ADL. Moreover, because body composition in Asian and Western populations is very different^13^ and lifestyle factors including kneeling and squatting in daily life also differ between Japan and Western countries, associations observed in whites may not be applicable to Japanese populations. If knee pain were shown to be associated with dependence in ADL among community-dwelling older populations, intervention in the form of exercise programs, for example, could be aimed at maintaining self-reliance in those with knee pain.

Therefore, the aim of this 3-year cohort study, in which the participants were highly representative of the target population, was to investigate the association of knee pain with dependence in ADL and mortality in community-dwelling older Japanese adults.

## METHODS

### Study population

This study is part of the Kurabuchi study, an ongoing community-based longitudinal study of aging that involves functional assessment of an older population in Kurabuchi Town, Takasaki City, a community approximately 100 km north of Tokyo, Japan. The details of the study have been described elsewhere.^14^^–^^16^ Briefly, from April through July 2005, trained public health nurses and local welfare commissioners conducted a home-visit health survey of residents aged 65 years or older, using a structured questionnaire with items on many aspects of health. Of the 1429 eligible residents, excepting those admitted to a hospital or an institution at the time of the survey, 1391 (97.3% of 1429) participated in the survey, and 1273 who were independent in ADL at baseline were followed as a cohort until September 2008, a period of approximately 3 years. Eight subjects moved out of the study area during follow-up; therefore, the associations between knee pain and the study outcomes were analyzed in 1265 residents (573 men, 692 women, follow-up rate: 99.4%). The median (range) age at baseline was 74 (65–97) years in men and 75 (65–98) years in women. The study was approved by the Medical Ethics Committee of the Keio University School of Medicine, Tokyo, Japan.

### Evaluation of knee pain

Presence of knee pain was assessed using the following question in the home-visit survey, “Have you had pain in the last year in or around the knee?,”^17^ with answer choices of “never,” “occasionally,” “often,” and “always.” This simple question seemed more suitable in a community setting than a complicated disease-specific instrument for measurement of knee pain, such as the Western Ontario and McMaster Universities Osteoarthritis index (WOMAC).^18^ Moreover, the English version of this question has been validated,^18^ although the Japanese version has not. In addition, to determine if subjects had gone to a physician for treatment of their knee pain, we asked the question, “Have you had a medical consultation regarding knee pain in the last year?,” with answer choices of “yes” or “no.”

### Outcome measurements

We defined dependence in ADL as either admission to a nursing home or need of assistance at home during the follow-up period. The latter was defined as long-term care (LTC) eligibility or a need for help in any of 6 basic ADL items on the Katz Index of Independence in ADL.^19^

LTC eligibility is a requirement for receiving LTC insurance services in Japan, which began in 2000. In this study, any of the 7 levels of LTC insurance services was considered LTC-eligible. However, not all residents who are dependent in ADL apply for LTC insurance services. We added the Katz ADL measures to address this possibility. The Katz ADL is based on self-reported performance levels for 6 basic ADL items (bathing, dressing, toileting, transferring, continence, and feeding), each of which has 3 answer options (without help, with partial help, with help). On the Katz ADL, dependence in ADL is defined as a need for partial or full help in performing any of the 6 items.

Information on death, nursing home admission, and LTC eligibility was obtained from the Kurabuchi Branch Office of Takasaki City Hall. Information on Katz ADL was obtained from repeat face-to-face home interviews conducted every year (in 2006, 2007, and 2008), and occurrence of ADL decline in any year was defined as an ADL decline.

### Covariates

Information on age, sex, marital status (married vs widowed/separated/single), education (junior high vs high school or higher), support by relatives, neighbors, and friends (yes vs no), smoking status (current vs former/never), alcohol drinking (current vs former/never), and current/past history of major diseases, including stroke, myocardial infarction/angina, chronic obstructive pulmonary disease, diabetes mellitus, and cancer (summary answer of yes or no), was also collected at the baseline survey, because these factors have been reported to be related to the study outcomes.^8^^,^^20^

### Statistical analyses

Stata 10.0 (Stata Corporation, College Station, TX, USA) was used for all analyses. Analyses were carried out by first conducting crude analyses, followed by multivariate analyses using logistic regression models. The initial basic model included only age (as a continuous variable) and sex. Then, the second and final models were constructed by adding marital status, education, smoking status, and current/past history of major diseases. There was no interaction between knee pain and medical consultation, and this was included in the model as an independent variable. Support by relatives, neighbors, and friends and alcohol drinking did not confound the observed association (the effect on the estimate was less than 10%^21^) and were thus not included in the model. Because there was no interaction by sex, all analyses were carried out with combined data for men and women. This analytic method was repeated for dependence in ADL, for the single outcome of mortality, and for the composite outcome of dependence in ADL and death. In the analysis of dependence in ADL, residents who died during the follow-up period were excluded. Finally, we analyzed each component of ADL dependence, including institutionalization, LTC eligibility, and the 6 items on the Katz basic ADL. Odds ratios (ORs) and 95% confidence intervals (CIs) were used to describe the strength of associations. Goodness of fit of all logistic models was assessed and confirmed by using the Hosmer–Lemeshow goodness-of-fit statistic (*P* values, 0.604–0.761).^22^

## RESULTS

The characteristics of the study population by knee pain status are shown in Table 1. A total of 667 (52.7%), 303 (24.0%), 128 (10.1%), and 167 (13.2%) participants reported never, occasionally, often, and always having knee pain, respectively. Furthermore, among the latter 3 groups, 28.1%, 52.3%, and 71.3%, respectively, reported seeking treatment from a physician for their knee pain. Participants who always had knee pain tended to be older, female, unmarried, and less educated than those in other categories. There was no difference among groups in history of major diseases, smoking status, or alcohol drinking.

**Table 1.** Characteristics of the study population

	Knee pain				*P* value^a^
	
	Never (*n* = 667)	Occasionally (*n* = 303)	Often (*n* = 128)	Always (*n* = 167)	
	No. (column %)	No. (column %)	No. (column %)	No. (column %)	
Age group, yrs					
65–69	198 (29.7)	63 (20.8)	14 (10.9)	13 (7.8)	<0.001
70–79	315 (47.2)	153 (50.5)	69 (53.9)	87 (52.1)	
≥80	154 (23.1)	87 (28.7)	45 (35.2)	67 (40.2)	
Sex					
Male	337 (50.5)	131 (43.2)	50 (39.1)	55 (32.9)	<0.001
Female	330 (49.5)	172 (56.8)	78 (60.9)	112 (67.1)	
Marital status					
Married	489 (73.6)	193 (64.3)	83 (65.9)	100 (60.6)	0.001
Widowed/separated/single	175 (26.4)	107 (35.7)	43 (34.1)	65 (39.4)	
Education					
Junior high school	502 (75.7)	241 (80.6)	99 (78.0)	144 (86.8)	0.014
High school or higher	161 (24.3)	58 (19.4)	28 (22.0)	22 (13.3)	
Consultation due to knee pain					
Yes	0 (0.0)	85 (28.1)	67 (52.3)	119 (71.3)	<0.001
No	659 (100.0)	217 (71.9)	61 (47.7)	48 (28.7)	
History of major disease^b^					
Yes	177 (26.7)	84 (27.9)	41 (32.3)	52 (31.5)	0.448
No	485 (73.3)	217 (72.1)	86 (67.7)	113 (68.5)	
Current smoking					
Yes	164 (24.6)	78 (25.8)	25 (19.7)	36 (21.7)	0.482
No	503 (75.4)	224 (74.2)	102 (80.3)	130 (78.3)	
Current alcohol drinking					
Yes	238 (36.2)	118 (39.3)	41 (32.5)	45 (27.8)	0.079
No	419 (63.8)	182 (60.7)	85 (67.5)	117 (72.2)	

^a^On χ^2^ test.

^b^Stroke, myocardial infarction/angina, chronic obstructive pulmonary disease, diabetes mellitus, or cancer.

Due to missing values, the totals of stratified subgroups are not equal.

Table 2 summarizes the associations of knee pain with the study outcomes. During the 3-year follow up, 109 participants died and 126 became dependent in ADL; thus, 235 in total (18.6% of 1265) experienced a study outcome. As the frequency of knee pain increased, the odds of being dependent in ADL increased. Those who always had knee pain were almost twice as likely to experience dependence in ADL as those who never had knee pain, even after adjustment for potential confounders (adjusted OR, 1.98; 95% CI, 1.03–3.83). Always having knee pain was associated with the composite outcome of ADL dependence and death in the crude analysis; however, after adjusting for covariates, the odds ratio became statistically insignificant. Knee pain was not associated with mortality. Requesting treatment from a physician for knee pain was not related to any outcome in this study population.

**Table 2.** Association of knee pain with dependence in activities of daily living (ADL) and death

	No. (%)	Crude OR (95% CI)	Age- and sex-adjusted OR (95% CI)	Multivariate-adjusted OR^a,b^ (95% CI)
Dependence in ADL^c^				
Never	57/609 (9.4)	1.00	1.00	1.00
Occasionally	22/279 (7.9)	0.83 (0.50–1.39)	0.61 (0.35–1.06)	0.64 (0.36–1.16)
Often	14/116 (12.1)	1.33 (0.71–2.47)	0.83 (0.42–1.63)	0.89 (0.42–1.88)
Always	33/152 (21.7)	2.69 (1.67–4.31)	1.84 (1.10–3.08)	1.98 (1.03–3.83)
Death				
Never	58/667 (8.7)	1.00	1.00	1.00
Occasionally	24/303 (7.9)	0.90 (0.55–1.48)	0.74 (0.44–1.26)	0.75 (0.43–1.33)
Often	12/128 (9.4)	1.09 (0.57–2.09)	0.81 (0.40–1.63)	0.76 (0.35–1.67)
Always	15/167 (9.0)	1.04 (0.57–1.88)	0.83 (0.44–1.56)	0.72 (0.32–1.61)
Death and dependence in ADL				
Never	115/667 (17.2)	1.00	1.00	1.00
Occasionally	46/303 (15.2)	0.86 (0.59–1.25)	0.64 (0.42–0.97)	0.67 (0.43–1.04)
Often	26/128 (20.3)	1.22 (0.76–1.97)	0.84 (0.50–1.43)	0.85 (0.47–1.52)
Always	48/167 (28.7)	1.94 (1.31–2.86)	1.45 (0.94–2.23)	1.46 (0.84–2.54)

Abbreviations: CI, confidence interval; OR, odds ratio.

^a^Model included age, sex, marital status, education, medical consultation, current/past history of major disease, and smoking.

^b^Due to missing data, only 1125 subjects for dependence in ADL and 1227 for death or composite outcome were included in the analyses.

^c^Participants who died during follow-up were excluded from this analysis.

The association between knee pain and each component of dependence in ADL was further examined (Figure). Among 126 subjects who were dependent in ADL, 36 were institutionalized. Of the remaining 90 who needed assistance at home, 76 were LTC-eligible and 48 were judged as dependent using the Katz index (35 in bathing, 27 in dressing, 17 in transferring, 18 in toileting, 27 in continence, and 7 in feeding, with some participants dependent in more than 1 activity). In this analysis of a smaller number of event occurrences, “never,” “occasionally,” and “often” were grouped together because there was no difference in the risk of ADL dependence, as shown in Table 1. As compared with never/occasionally/often having knee pain, always having knee pain was associated with LTC eligibility (adjusted OR, 2.61; 95% CI, 1.34–5.07) and dependence in bathing (2.71, 1.08–6.82), dressing (3.65, 1.30–10.28), and transferring (5.90, 1.78–19.50).

**Figure.**
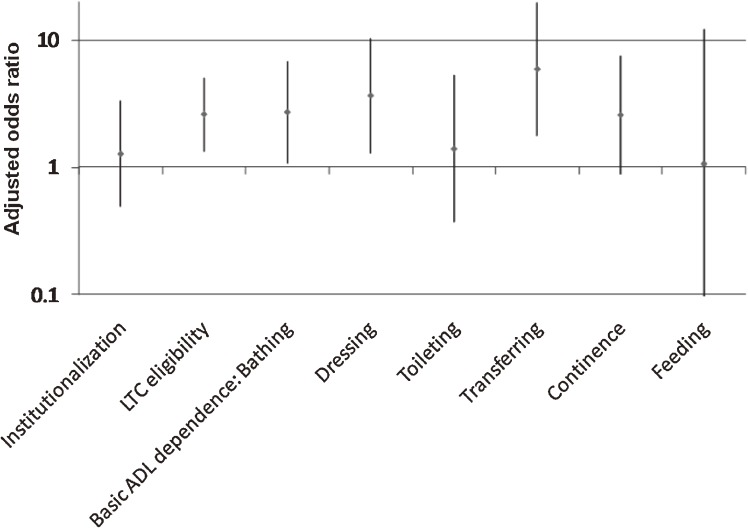
Multivariate-adjusted odds ratios and 95% confidence intervals for always having knee pain as compared with never/occasionally/often having knee pain were plotted using a logarithmic scale, after adjusting for age, sex, marital status, education, medical consultation, current/past history of major disease, and smoking. Participants who died during follow-up were excluded. Additionally, participants who were institutionalized during follow-up were excluded from the estimation of odds ratios of LTC eligibility and basic ADL dependence on the Katz ADL.

## DISCUSSION

Knee pain was clearly associated with dependence in ADL, but not with the single endpoint of mortality or the composite outcome of dependence in ADL and death. To minimize bias, much attention was placed on attaining high representation and a high follow-up proportion. Another strength of this study is that many potential confounders were included in the analysis. To the best of our knowledge, this is the first community-based epidemiological study of the association of knee pain with dependence in ADL and mortality.

Participants who reported always having knee pain were more likely to become ADL-dependent. This result was in line with earlier studies, which reported a clear association of knee pain with functional decline in ambulatory activities.^6^^,^^23^ Constant pain, regardless of whether the individual seeks medical treatment, can be a good marker of future dependence in ADL. This marker requires only one, simple question and can be very useful in both clinical and community settings. Further analyses revealed that knee pain was associated with need of assistance at home, but not with institutionalization. Bathing and transferring undoubtedly require mobility/muscular strength of the lower extremities; dressing may involve walking to get clothes from a closet.^19^ Thus, the association of a need for assistance in these activities with knee pain was expected. It should also be noted that this is, to the best of our knowledge, the first report to assess the relationship between LTC eligibility and knee pain. Information revealed by our study on future care needs should be useful for caregivers. However, because we were unable to consider multiple comparisons in this analysis, caution is warranted in interpreting the present results.

Knee pain was not associated with an increased risk of death. Although the lack of an association between knee pain and mortality has previously been reported,^24^ such a result is counterintuitive because ADL dependence is generally a good predictor of mortality.^25^^,^^26^ Indeed, in the present study, knee pain tended to be inversely associated with mortality. A possible explanation for this finding is that 71% of subjects with knee pain were regularly consulting physicians for this complaint, thus increasing the chance that risk factors for mortality, such as hypertension and hyperlipidemia, would be detected and treated early. In any event, further studies with longer follow-up periods are needed to elucidate the association between knee pain and death.

The present study illustrates how knee pain plays a key role in the self-reliance of older adults and highlights the importance of preventing knee pain. The effect of exercise in alleviating knee pain has been reported.^27^^,^^28^ Enlightenment of the general public regarding the importance of preventing musculoskeletal problems, and knee pain in particular, should be continued and even expanded. Our findings also suggest that intervention programs that foster self-reliance should be aimed at older people with knee pain. In fact, exercise programs for reducing knee pain are cited in the preventive approach manual issued in conjunction with LTC insurance services. However, the checklist used for screening candidates does not include the presence of knee pain itself, although it does include mobility limitation.

A limitation of this study is that knee pain was assessed at only 1 time point, and the course of pain was not evaluated. In a Dutch study, 44% of patients presenting to a general practice with knee complaints recovered within 12 months.^29^ The difference in knee pain prognosis after treatment is an important issue that requires further study. Another limitation in the present study is the possibility of confounding by unmeasured factors, such as body mass index and occupation. Overweight in particular can be a risk factor for future ADL dependence,^30^^,^^31^ so further discussion of this variable is needed. If overweight is the result of inactivity due to knee pain, it should not be included in models as a covariate. However, Kondo et al suggested that overweight precedes knee OA and is a predictor of stair-climbing limitation rather than an outcome of knee OA.^11^ Because overweight is sometimes associated with ADL dependence through pathways other than knee pain, eg, hip pain or ankle pain, adjustment for overweight is necessary, and the OR observed in this study might thus have been overestimated. However, Lamb et al suggested that overweight modified the association between knee pain and mobility limitation.^32^ Obviously, in such a case, analysis stratified by BMI category would be appropriate. Furthermore, relatively crude classification of a covariate such as education category might cause residual confounding. Also, we could not determine whether knee pain resulted from OA, rheumatoid arthritis, or other causes.

In conclusion, after nearly perfect follow-up of participants that were highly representative of the target population, we found that knee pain was clearly associated with dependence in ADL, and with a need for assistance at home (LTC-eligibility, bathing, dressing, and transferring) in particular, but not with the single endpoint of mortality or the composite outcome of dependence in ADL and death.
